# Radiograph-Based Deep Learning Model to Support Finger Joint Selection for Ultrasound Examination in Rheumatoid Arthritis

**DOI:** 10.3390/diagnostics16111689

**Published:** 2026-05-29

**Authors:** Youngjae Park, Keum San Chun, Seungeun Lee, Joon-Yong Jung, Sungwon Lee, Hyemin Park, Tuan Dinh Le, Hyeondeok Choi, Wan-Uk Kim

**Affiliations:** 1 Department of Rheumatology, Seoul St. Mary’s Hospital, College of Medicine, The Catholic University of Korea, Seoul 06591, Republic of Korea; 2 Department of Radiology, Seoul St. Mary’s Hospital, College of Medicine, The Catholic University of Korea, Seoul 06591, Republic of Korea; 3 Visual Analysis and Learning for Improved Diagnostics (VALID) Lab, College of Medicine, The Catholic University of Korea, Seoul 06591, Republic of Korea

**Keywords:** rheumatoid arthritis, hand, radiography, inflammation, deep-learning

## Abstract

**Background/Objectives:** Ultrasound is the standard imaging modality to evaluate the inflammatory changes in hand joints of rheumatoid arthritis (RA) patients. However, it is operator-dependent and takes a long time to examine. In this study, we developed a radiograph-based deep learning (DL) model to support prioritization of finger joints for ultrasound (US) examination in RA patients. **Methods:** In this retrospective study, hand radiographs from RA patients who underwent same-day US examination of bilateral finger joints were analyzed. A DL model was developed using hand radiographs from 270 patients (2043 finger joints) to estimate joint-level likelihood of inflammatory activity. US findings served as the reference standard for model training, while clinical findings of joint tenderness and swelling were incorporated as additional tabular inputs. Model performance was evaluated in a temporal-split test cohort consisting of 40 patients (270 joints) and compared with the performance of a clinical-only logistic regression model based on joint tenderness and swelling. **Results:** In the test set, the DL model demonstrated higher sensitivity (82.1% vs. 38.5%), negative predictive value (96.8% vs. 90.3%), and F1-score (69.6% vs. 48.4%) than the clinical-only model. Although the area under the receiver operating characteristic curve did not differ significantly between models (*p* = 0.43), precision–recall (PR) analysis showed superior performance of the DL model, with a higher area under the PR curve (0.625 vs. 0.540). At the threshold maximizing the F1-score, DL-assisted triage reduced the number of finger joints selected for US examination by approximately 80%. **Conclusions:** A radiograph-based DL model can support efficient prioritization of finger joints for US examination in RA, offering a practical approach to enhance joint-level US triage in routine clinical practice.

## 1. Introduction

Rheumatoid arthritis (RA) is a chronic autoimmune disorder that primarily affects the synovial joints and further damages the joint structure. As complete remission of RA is difficult to achieve, the treatment goal is often to maintain low disease activity. Disease activity is typically monitored through regular blood tests and various imaging modalities such as radiographs, ultrasound (US), and magnetic resonance imaging (MRI) [1]. Radiographs are traditionally used to evaluate structural damage, such as bone erosions and joint space narrowing. At the same time, US and MRI are more effective for detecting synovitis and other inflammatory changes [2,3]. As each imaging modality provides distinct information regarding disease pathology, leveraging all three imaging modalities is important for comprehensive assessment.

In clinical practice, RA patients who present with joint pain, stiffness, or swelling typically undergo both hand radiographs and US, with hand radiographs often performed first to assess structural changes, followed by US for evaluation of active inflammation. Although US is a sensitive modality for detecting synovial inflammation, including subclinical inflammation, its routine use is limited by operator dependency and the time required for comprehensive joint assessment [4,5]. To reduce variability in US assessment, standardized scoring systems such as the OMERACT–EULAR Synovitis Scoring system have been proposed [6,7]. However, there remains no consensus regarding the optimal number or selection of joints to be examined, with existing protocols ranging from extended to reduced joint sets [8,9]. Moreover, prior randomized controlled trials have reported heterogeneous results regarding the clinical benefit of routine US-guided management, despite the prognostic value of ultrasound-detected inflammatory activity for structural progression and disease flares [8,9,10,11]. Taken together, these considerations suggest a need for a more objective and efficient approach to support decisions on joint selection for US examination.

Imaging findings in RA often require integration with clinical symptoms and physical examination findings, making close collaboration between clinicians and radiologists essential for accurate imaging-based assessment of RA [12]. Recent advances in artificial intelligence (AI) may further support this collaborative workflow by enabling automated assessment and risk stratification approaches in RA imaging. Machine learning and deep learning (DL) techniques have been applied to a wide range of tasks, including disease classification, prediction of treatment response, and monitoring of disease progression, using data from electronic health records, serologic markers, and medical imaging [10,13,14]. In medical image analysis, a previous study demonstrated the feasibility of convolutional neural network (CNN)-based automated diagnosis of RA using hand radiographs for identifying RA-related imaging abnormalities [15]. Furthermore, CNN-based DL models have shown considerable promise in the automated assessment of structural damage in RA, particularly for hand analysis. One previous study aimed to improve the sensitivity of radiographic progression assessment by quantitatively detecting subtle joint space narrowing that may be difficult to recognize visually [16]. Another study developed an automated modified total Sharp/van der Heijde score (mTSS)-based framework incorporating joint localization and classification for systematic radiographic scoring [17]. More recently, transformer-based and contextual comparison models have been introduced to further improve automated assessment of joint-level bone erosions and joint space narrowing [18,19].

Hand radiography is a practical and scalable modality for DL-based analysis, because of its standardized acquisition, short imaging time, and high reproducibility. However, despite these advantages, the potential role of radiographs in guiding joint selection for subsequent US examination has received limited attention. We hypothesized that hand radiographs contain visual features related to synovial inflammation that are complementary to clinical examination, and that integrating radiographic features with basic findings of tenderness and swelling could improve identification of joints with active inflammation compared with clinical assessment alone. Therefore, we aimed to develop a DL model that leverages hand radiographs to estimate joint-level likelihood of US-detected inflammatory activity to support US triage by prioritizing target joints for further evaluation in RA patients.

## 2. Materials and Methods

### 2.1. Study Design and Datasets

This retrospective study was approved by the institutional review board of The Seoul St. Mary’s Hospital (approval number: KC24RISI0071). The requirement for written informed consent was waived due to the retrospective nature of the study and the use of anonymized data. The full study protocol is not publicly available, and details of the study design and analysis are provided in the Methods section of this manuscript. This study was designed to primarily evaluate joint-level US triage performance of the DL model, with particular emphasis on sensitivity and negative predictive value (NPV) given the screening-oriented nature of the proposed approach. Secondary analyses included receiver operating characteristic (ROC) and precision–recall (PR) analyses comparing the DL model with a clinical-only model, as well as threshold-based evaluation of reduction in the number of finger joints selected for US examination.

**Figure 1:**
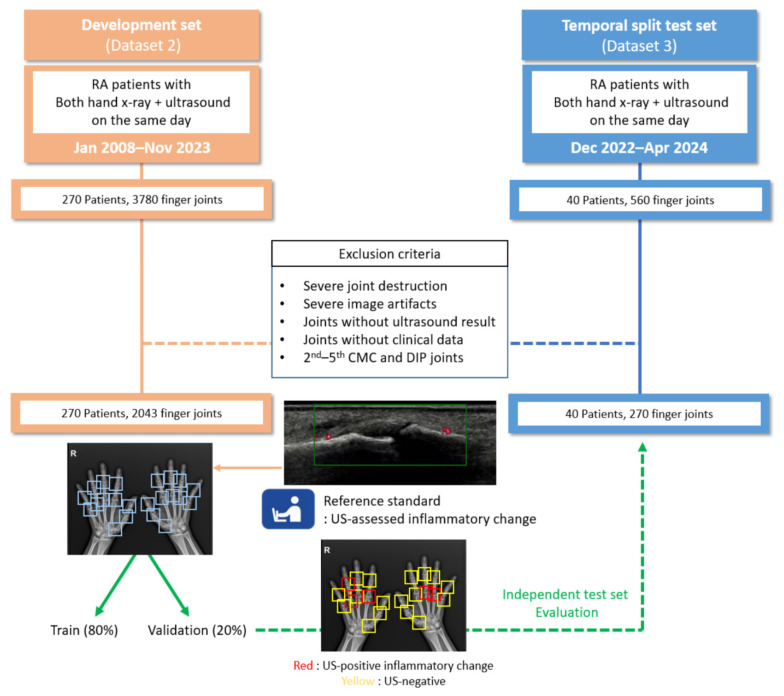
Workflow for model development and evaluation. The DL model was developed using a development set (Dataset 2) and evaluated on an independent temporal test set (Dataset 3), including RA patients who underwent same-day hand radiography and ultrasound. US-assessed inflammatory change was used as the reference standard for joint-level classification.

Hand radiographs from two institutions were grouped into three datasets according to their role in model development and evaluation. Dataset 1 consisted of a publicly available hand radiograph dataset obtained from the V.A. Nasonova Research Institute of Rheumatology and was used exclusively for training the automated joint localization step [18]. This dataset was selected to provide sufficient anatomical variability for robust landmark learning and was not used for inflammation labeling or performance evaluation. Datasets 2 and 3 were retrospectively collected from The Seoul St. Mary’s Hospital between January 2008 and April 2024 (Figure 1). These datasets included radiographs from consecutive RA patients who visited the rheumatology outpatient clinic with joint pain or swelling in finger joints. All included patients fulfilled the 2010 ACR/EULAR RA classification criteria and underwent both hand radiographs (FD-X and Fluorospot Compact FD, Siemens Healthineers, Erlangen, Germany; Digital Diagnost, Philips Healthcare, Best, The Netherlands) and US examinations (Samsung Medison Co. Ltd., Seoul, Republic of Korea) of bilateral finger joints on the same day. No formal sample size calculation was performed. The sample size was determined by the number of eligible patients available during the study period, consistent with prior studies evaluating machine learning models for joint-level imaging analysis. Based on the acquisition date, radiographs were temporally divided in December 2022, resulting in 270 radiographs for Dataset 2 (from January 2008 to November 2022) and 40 radiographs for Dataset 3 (from December 2022 to April 2024). Dataset 2 was used for model development and internal validation, while Dataset 3 served as an independent temporal test set. All participants of Datasets 2 and 3 were Asian, as documented in the electronic medical records (EMRs).

### 2.2. Joint-Level Inclusion and Exclusion Criteria

Joint-level analysis was performed for Datasets 2 and 3. The following exclusion criteria were set for the joint-wise approach: (1) severe structural joint damage, (2) severe image artifacts, (3) absence of US result report, (4) missing clinical data on tenderness and swelling, and (5) distal interphalangeal (DIP) joints and second to fifth carpometacarpal (CMC) joints.

Severe structural joint damage was defined as complete joint space loss or subluxation exceeding 50% of the long bone shaft alignment on radiographs. Joints meeting this criterion were excluded to reduce potential confounding between chronic structural changes and active inflammatory findings on US. Clinical data on joint tenderness and swelling were required because these basic physical examination findings were incorporated into both the DL model and the clinical-only model; therefore, joints lacking this information were excluded. Joint tenderness and swelling were recorded as binary variables (present or absent) based on physical examination findings documented in the EMR. DIP joints were excluded because they are infrequently involved in rheumatoid arthritis, whereas second to fifth CMC joints were excluded due to substantial joint overlap and superimposition on hand radiographs, which limit reliable joint-level assessment. After applying these exclusion criteria, the final datasets comprised 2043 finger joints in Dataset 2 and 270 finger joints in Dataset 3.

### 2.3. Ultrasound Reference Standard

US-based labels for finger joints in Datasets 2 and 3 were assigned by a radiologist (S.L., 7 years of experience in musculoskeletal radiology) and a rheumatologist (Y.P., 8 years of experience in rheumatology). The reviewers evaluated US images and corresponding reports using the institutional picture archiving and communication system (PACS) and EMR, and were blinded to the results of the clinical information. Based on these evaluations, each joint was classified in a binary manner (positive or negative) for the presence of inflammation. A positive label was assigned when two or more of the following findings were present: synovitis, joint effusion, or a positive power Doppler signal. A negative label was assigned when less than two of these findings were observed. Discrepancies between image interpretation and written reports were resolved through consensus review.

### 2.4. Deep Learning Model Development

#### 2.4.1. Automated Joint Localization

Automated joint localization was performed using DeepLabCut (DLC) algorithm, a landmark-based deep learning approach for anatomical point detection [20]. Finger joint center coordinates were manually annotated by a radiologist (H.P., 4 years of experience in musculoskeletal radiology) and used to train the localization model. The trained model was applied to identify joint centers for subsequent image cropping. Detailed information regarding model architecture, annotation procedures, and localization performance is provided in Supplementary Material S1.

#### 2.4.2. US Triage Model Development and Validation

DL model was trained using a pipeline consisting of image preprocessing, feature extraction, multimodal feature fusion, and binary classification. During preprocessing, pixel value normalization, contrast enhancement, and conservative data augmentation techniques were applied to standardize radiographs acquired from different machines. Joint-centered images were cropped to 224 × 224 pixels based on the coordinate identified during the joint localization step. To address class imbalance, minority-class samples were oversampled during training.

**Figure 2:**
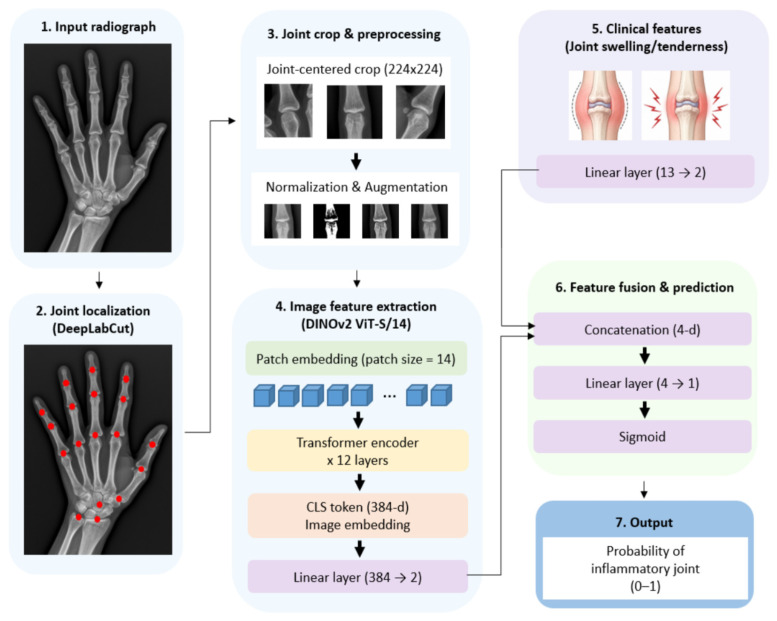
Overview of the proposed multimodal deep learning pipeline for prediction of ultrasound (US)-detected finger joint inflammation. Joint-centered radiographic images were processed using a pretrained DINOv2 vision transformer backbone, while clinical variables including joint swelling, tenderness, and joint type were incorporated as tabular features. Image-derived and clinical features were fused for final prediction of US-detected inflammation.

Feature extraction was performed using a pretrained DINOv2 vision transformer model (ViT-Small/14), consisting of a 14 × 14 patch-embedding layer and 12 transformer encoder blocks with GELU activation functions [21]. Given the limited sample size, the pretrained backbone was frozen and only the downstream classification layers were fine-tuned. Clinical variables were incorporated as additional tabular input features and fused with image-derived features to generate a prediction probability for US-detected inflammation (Figure 2). This probability was subsequently converted into a binary classification using US-derived labels as reference standards.

The model was trained using binary cross-entropy loss with the AdamW optimizer (learning rate, 5 × 10^−4^; batch size, 64) for up to 300 epochs. Validation loss was monitored during training, and the model state with the lowest validation loss was retained as the final checkpoint, effectively implementing early stopping. The final model was trained on Dataset 2 with internal validation and subsequently evaluated on the temporally held-out Dataset 3. Further information on model development is provided in Supplementary Material S2.

### 2.5. Statistical Analysis

Model performance was evaluated on Dataset 3 using the accuracy, sensitivity, specificity, positive predictive value (PPV), NPV, and F1-score. The operating threshold was primarily determined as the value maximizing the F1-score on the test set, reflecting a balanced trade-off between precision and recall. The performance of the DL model was compared with that of a clinical-only logistic regression model incorporating joint tenderness and swelling using ROC curves and PR curves. Differences in area under the ROC curve (AUC) between the two models were assessed using the DeLong test. This comparison was designed to evaluate the incremental value of an AI-assisted strategy over symptom-based clinical assessment alone for joint-level US triage. Statistical analysis was performed with use of R (version 4.0.0.; R foundation for Statistical Computing, Vienna, Austria) and Python (version 3.10.; Python Software Foundation, Beaverton, OR, USA).

To reflect different clinical priorities in joint-level US triage, confusion matrices were generated at multiple selected probability thresholds to characterize threshold-dependent classification performance. Probability thresholds were systematically varied to examine the trade-offs between safety and efficiency, defined as the ability to safely exclude joints unlikely to demonstrate US-detected inflammatory activity (as reflected by sensitivity and NPV) and the proportion of finger joints that could be excluded from subsequent US evaluation.

## 3. Results

The demographics and image distribution of patients in Datasets 1, 2, and 3 are summarized in Table 1. US-detected inflammatory joints accounted for 245 of 2043 joints in Dataset 2 and 38 of 270 joints in Dataset 3. Automated joint localization achieved sufficient accuracy to enable consistent joint-centered image cropping, with detailed performance results provided in Supplementary Figures S1 and S2.

The performance of the DL model on Dataset 3 is presented in Table 2 and compared with that of a clinical-only model based on joint tenderness and swelling. The DL model demonstrated a higher sensitivity (82.1% vs. 38.5%) and NPV (96.8% vs. 90.3%) compared with the clinical-only model, while showing lower specificity (90.9% vs. 96.5%) and PPV (60.4% vs 65.2%).

**Figure 3:**
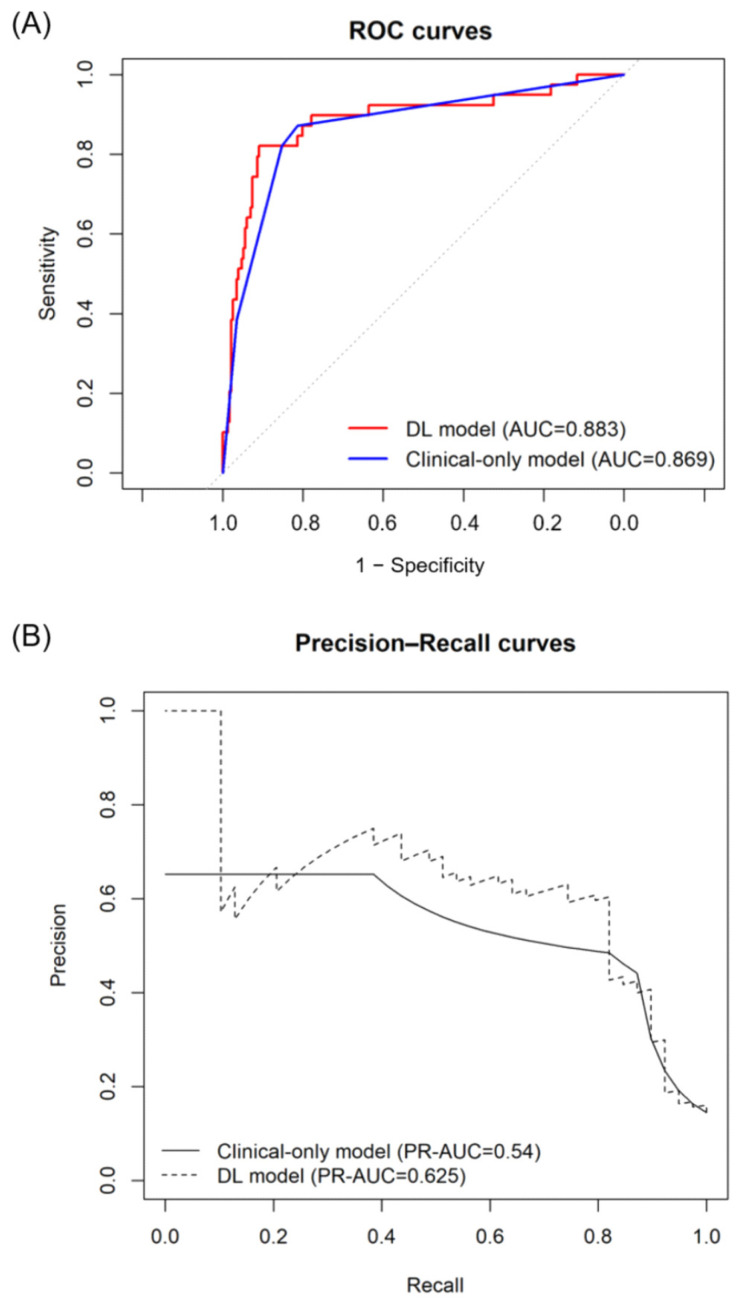
Receiver operating characteristic (ROC) and precision–recall (PR) curves comparing the deep learning (DL) model and the clinical-only model in the test set. (**A**) ROC curves. (**B**) Precision–recall curves.

The DL model achieved a higher F1-score than the clinical-only model (69.6% vs. 48.4%). ROC analysis showed no significant difference in AUC between the two models (*p* = 0.43; Figure 3). However, despite comparable ROC AUCs, the DL model demonstrated superior performance on PR curve analysis, achieving a higher PR-AUC than the clinical-only model (0.625 vs. 0.540; Figure 3). This improvement reflected higher precision across a wide range of recall levels, particularly within the clinically relevant intermediate recall range (0.3–0.8).

**Figure 4:**
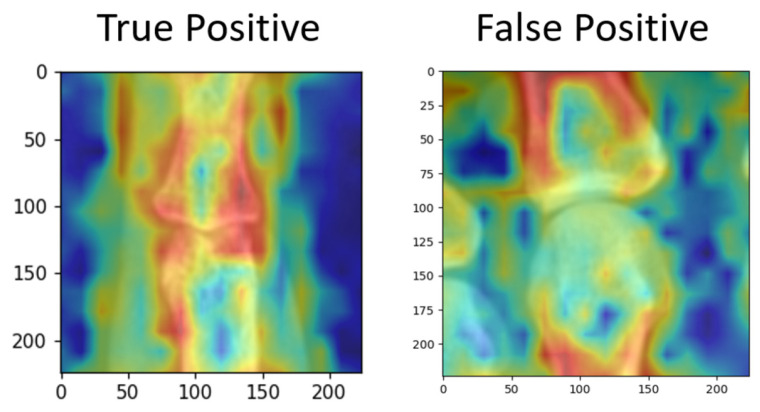
Representative heatmaps of true positive and false positive predictions in the finger joint. Red and yellow regions indicate areas receiving greater model attention, whereas blue regions indicate areas with relatively low attention. In true positive case, the attended regions focused on the joint areas and overlying soft tissue regions. In contrast, the attended regions were sparse in the joint areas and predominantly focused on bony structures in false positive case.

Class activation heatmaps were used to visualize differences in model attention between true positive and false positive predictions (Figure 4). In true-positive cases, the DL model predominantly focused on the joint region and adjacent periarticular soft tissues. In contrast, false positive cases showed sparse attention within the joint space, with greater emphasis on bony structures.

**Figure 5:**
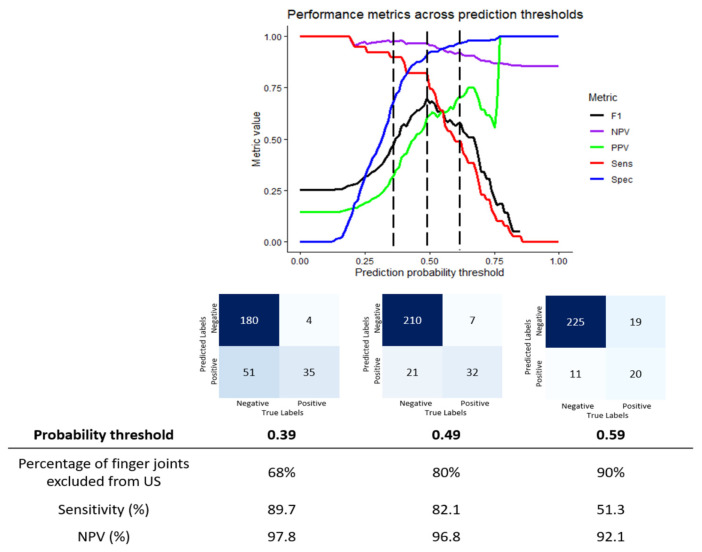
Threshold-dependent changes in the number of finger joints excluded from ultrasound (US) examination, sensitivity, and negative predictive value (NPV), with the operating threshold defined by the maximum F1-score.

Adjustment of the probability threshold revealed a clear trade-off between safety and efficiency in joint-level US triage (Figure 5). As the threshold increased, a larger proportion of finger joints could be excluded from US examination, thereby improving work efficiency. However, this was accompanied by reduced sensitivity and NPV, indicating an increased risk of missed inflammatory findings. Conversely, lower probability thresholds prioritize higher sensitivity and NPV, enabling safer exclusion of joints at the cost of selecting a greater number of joints for US examination.

Using confusion matrices derived from the test set, we quantified the number of joints that could potentially be bypassed during US examination at selected probability thresholds (Figure 5). When cases predicted as negative were excluded, the estimated reduction in the number of joints reached 80% at the probability threshold corresponding to the highest F1-score. At this threshold, AI-assisted triage reduced the average number of finger joints selected for US examination in both hands to six of 22 finger joints, which is comparable to the conventional reduced 7-joint set.

## 4. Discussion

In this study, we developed the DL model to support US triage by automatically identifying finger joints with suspected inflammatory change on hand radiographs. The proposed model demonstrated reliable performance and outperformed a model based solely on clinical information. These results support the feasibility of using hand radiographs as a preliminary screening tool for selecting target joints for subsequent US examinations. By prioritizing joints likely to demonstrate inflammatory activity while excluding low-risk joints, the model has the potential to reduce US examination time and improve workflow efficiency.

Previous studies have developed DL models for detecting joint destruction in hand radiographs of RA patients [17,18,19]. However, developing DL models that can evaluate inflammatory change by focusing on the soft tissue area surrounding the joint space and capsule in hand radiographs is considerably more challenging than modeling osseous abnormalities. Even for experienced radiologists, early inflammatory changes are difficult to detect on hand radiographs alone [22,23]. As a result, prior DL studies targeting inflammatory changes in RA have primarily relied on US and MRI data [24,25,26]. These studies reported DL model accuracies ranging from 0.70 to 0.84. Similarly, our DL model, based on hand radiographs, achieved better accuracy of 0.88. This finding suggests that hand radiographs, despite their limited sensitivity for early inflammation, can serve as a meaningful and practical imaging modality for deep learning-based analysis, capturing imaging patterns relevant to joint-level inflammatory activity.

Building on this capability, our DL model can help prioritize finger joints that warrant US evaluation, potentially reducing examination time. Previous studies have explored various reduced joint sets corresponding to US examination protocols. However, selecting an optimal joint set remains challenging, and the reported clinical benefit of routine US-guided management has been inconsistent [8,9,11,27]. In routine clinical practice, decisions regarding US examination are often based on patients’ symptoms and physical examination findings such as tenderness or swelling, which are typically assessed in a binary manner and may limit joint-level risk stratification. By incorporating radiographic information from hand radiographs, our DL model generated more discriminative probability estimates and demonstrated improved F1-scores and PR-AUC compared with clinical assessment alone. Given that PR-AUC is particularly informative in class-imbalanced settings, these findings support the potential role of the radiograph-based DL model as an effective tool for US triage in RA.

Our DL model has several limitations. First, detecting inflammation flare-ups in chronic destructive joints is not applicable, as destructive joints were excluded from input data. Extension of the model to destructive joints and other anatomical regions, including the wrist, remains an important area for future investigation. Second, the model was fine-tuned to screen the target joints for subsequent US examination, and it is not suitable for the final diagnosis of inflammatory change in finger joints. Further prospective studies are needed to determine whether this triage-oriented approach can improve the efficiency and diagnostic yield of routine US examination workflows. Third, external validation was not conducted due to the limited availability of paired hand radiographs and US data that assess all finger joints in other institutions. Nonetheless, the model’s generalizability is supported because hand radiographs in Datasets 2 and 3 were acquired using three radiograph scanners. In addition, the temporally split test set primarily comprised images acquired from one scanner, which differed from the other two scanners used for most of the training data. This setup inherently tested the model’s robustness across devices and periods. Future multicenter external validation and extension to multimodal frameworks integrating radiographic features with MRI or laboratory markers may further improve generalizability and clinical applicability.

In conclusion, the proposed DL model demonstrated reliable performance in identifying finger joints with suspected inflammatory change on hand radiographs, supporting the feasibility of radiograph-based assessment as a US triage tool in patients with early RA. By providing joint-level information to prioritize targets for US examination, the model has potential to support more informed decision-making and streamline diagnostic workflows in routine clinical practice.

## Figures and Tables

**Table 1:** Patients’ demographics and distribution of ultrasound-based joint labels in Datasets 1, 2, and 3.

	Dataset 1	Dataset 2	Dataset 3
Age (median, range)	48 (20–62)	55 (19–87)	55 (27–80)
Sex (M:F)	50:280	51:219	3:37
No. of radiographs	330	270	40
No. of joints (US-negative:US-detected inflammation)	-	1798:245	232:38

**Table 2:** Performance comparison between the DL model and the clinical-only model for joint-level US triage in the test set. Model performance was evaluated using accuracy, sensitivity, specificity, positive predictive value (PPV), negative predictive value (NPV), and F1-score at the operating threshold that maximized the F1-score.

	DL Model	Clinical-Only Model
Joints (no.)	270	270
Prevalence (%)	14	14
Accuracy (%)	89.6	88.1
Sensitivity (%)	82.1	38.5
Specificity (%)	90.9	96.5
Positive predictive value (%)	60.4	65.2
Negative predictive value (%)	96.8	90.3
F1-score (%)	69.6	48.4
Area under the ROC curve ^†^	0.88	0.87

^†^ Receiver operating characteristic curve.

## Data Availability

The datasets analyzed during the current study are not publicly available due to institutional and ethical restrictions but are available from the corresponding author on reasonable request, subject to approval by the Institutional Review Board of Seoul St. Mary’s Hospital. The code used in this study is also available on reasonable request. Requests should be sent via email to the corresponding author.

## Supplementary Materials

The following supporting information can be downloaded at: https://www.mdpi.com/article/10.3390/diagnostics16111689/s1, Figure S1: Result of automated joint localization in hand and wrist joints using the DeepLabCut algorithm; Figure S2: Example of joint localization in a finger joint using the DeepLabCut algorithm.

## Abbreviations

The following abbreviations are used in this manuscript:

RA	Rheumatoid arthritis
US	Ultrasound
MRI	Magnetic resonance imaging
AI	Artificial intelligence
DL	Deep learning
CNN	Convolutional neural network
EMR	Electronic medical record
DLC	DeepLabCut algorithm
CMC	Carpometacarpal
MCP	Metacarpophalangeal
PIP	Proximal interphalangeal
DIP	Distal interphalangeal
RMSE	Root mean squared error
PPV	Positive predictive value
NPV	Negative predictive value
ROC	Receiver operating characteristic
PR	Precision–recall
AUC	Area under the curve
