# Supplementary material for: Radiograph-Based Deep Learning Model to Support Finger Joint Selection for Ultrasound Examination in Rheumatoid Arthritis
Source: Diagnostics (Basel). 2026 May 29;16(11):1689. doi: 10.3390/diagnostics16111689 (PMC13256136; doi:10.3390/diagnostics16111689)
Supplement: Supplementary file 1 [file diagnostics-16-01689-s001.zip › diagnostics-4279133-supplementary.pdf]

Supplementary material 1. Detailed description of the DeepLabCut (DLC) model architecture, annotation procedures, and model performance metrics for joint localization using hand radiographs

The DLC architecture is based on a pre-trained deep residual network (ResNet-50), which extracts image features through multiple hierarchical layers. It also incorporates deconvolutional layers to generate dense heatmaps for each region of interest (ROI). This landmark-based approach was preferred over bounding-box detectors (e.g., YOLO, Faster R-CNN) because joints in hand radiographs are small, densely clustered, and require high-precision localization. DLC allows accurate point-based detection of joint centers, enabling consistent cropping while reducing labeling effort and training time for anatomical tasks.

For the standard references of the joint locations, one radiologist (H.P., 3 year of experience in musculoskeletal radiology) manually annotated the center point of total 36 anatomical landmarks across bilateral wrist and finger joints in Dataset 1 and 2; carpometacarpal (CMC), metacarpophalangeal (MCP), proximal interphalangeal (PIP), and distal interphalangeal (DIP) joints. The DLC algorithm was subsequently trained using Dataset 1 with a 95% training fraction and a batch size of 8, with data augmentation performed using the default DLC strategy. The model's performance was tested on Dataset 2 using the root mean squared error (RMSE) metric.

Supplementary figure S1. Result of automated joint localization in hand and wrist joints using the DLC algorithm. Joint-wise RMSE values are shown, with an overall mean localization error of 0.46 ± 0.12 mm across all finger joints. In x-axis, hand joints were labeled using a combination of laterality, digit number, and joint name (e.g., R-3-MCP for the right third metacarpophalangeal joint). CMC: carpometacarpal joint, MCP: metacarpophalangeal joint;

PIP: proximal interphalangeal joint, DIP: distal interphalangeal joint.

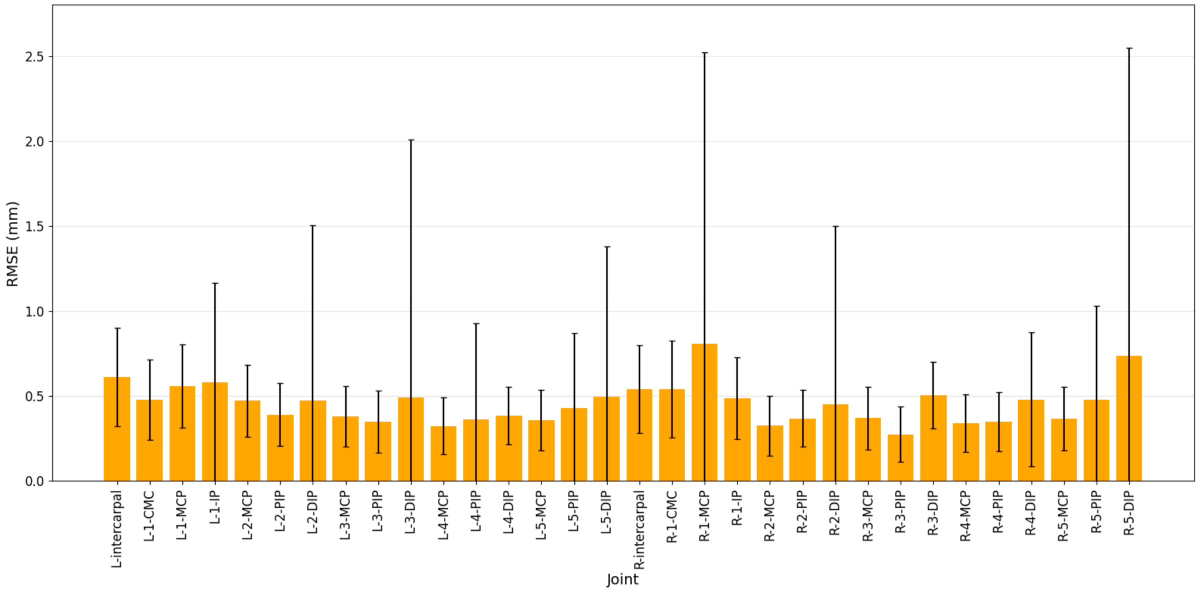

Supplementary figure S2. Example of joint localization in a finger joint using the DLC algorithm. The reference joint locations (cross marker) and predicted locations (dot) are closely aligned.

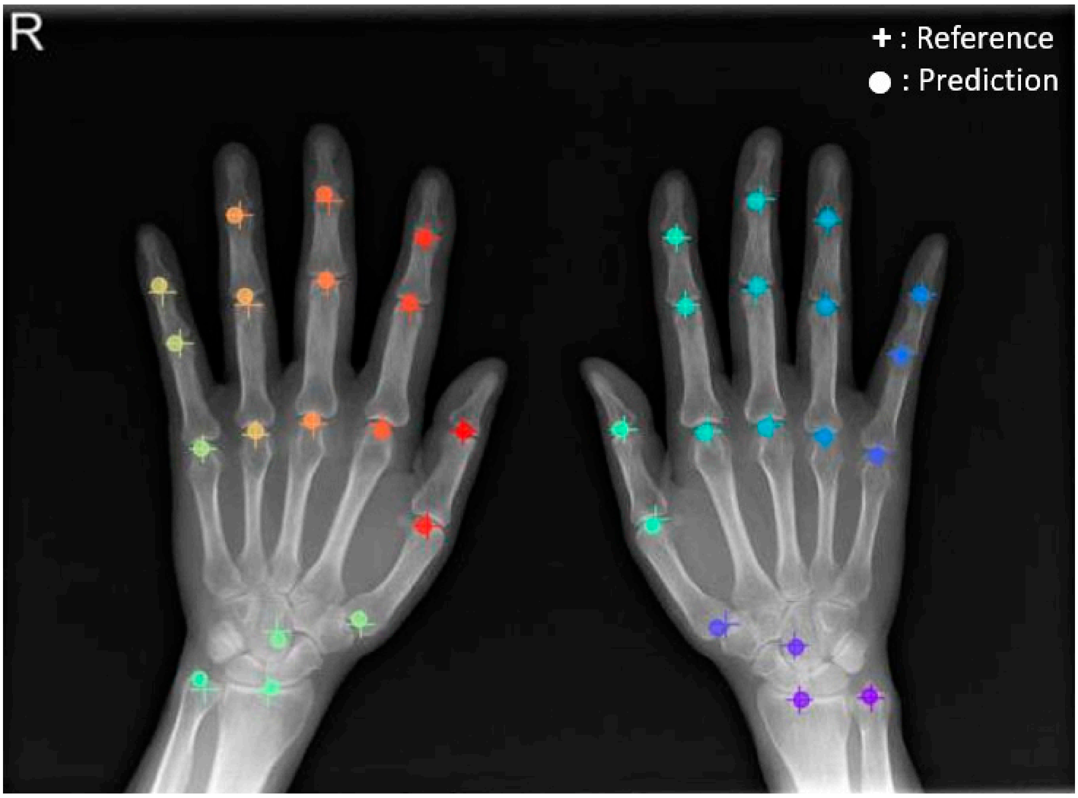

Supplementary material 2. Detailed description of the deep learning model framework and training procedure for joint inflammation detection using hand radiographs

The deep learning (DL) pipeline consisted of image preprocessing, feature extraction using a pretrained DINOv2 backbone, multimodal feature fusion with clinical variables, and binary classification for prediction of ultrasound (US)-detected inflammation in finger joints.

2.1 Image preprocessing

The image preprocessing step consisted of pixel value normalization, contrast enhancement, deterministic smoothing, image resizing, and channel conversion. Radiographs were acquired from three different machines with varying configurations, resulting in heterogenous image contrast across the dataset. To mitigate this variability, pixel values below the most frequent value, representing the background color, were set to zero, while pixel values above the most frequent value were normalized using min-max normalization. After the normalization, joint images were cropped to 224x224 pixels, centered at the coordinate identified during the automated joint localization step. Contrast enhancement was applied using Contrast Limited Adaptive Histogram Equalization (CLAHE; clipLimit = 2.0, tileGridSize = 8×8). Gaussian blurring (kernel size = 5×5, $\sigma = 3$) was deterministically applied to suppress sonsor noise while preserving periarticular soft tissue contrast. Finally, grayscale radiographs were converted to three-channel images by duplicating the single channel, in order to match the input requirements of the pretrained DINOv2 model.

2.2 Data augmentation and class balancing

Data augmentation techniques including random rotation (approximately ±1°) and additive Gaussian noise (standard deviation = 0.01, i.e., 1% intensity variation) were applied to the training dataset. To preserve subtle soft tissue features surrounding the joint,

augmentation magnitudes were intentionally kept conservative. After data augmentation, the number of positive and negative label cases was balanced by joint-wise oversampling of minority-class samples prior to training.

2.3 Model architecture

The pretrained DINOv2 model (ViT-Small/14) was used as the image feature extraction backbone. DINOv2 is a self-supervised vision transformer framework capable of learning robust and generalizable image representations. Given the limited sample size, the pretrained DINOv2 backbone was frozen, while the downstream classification layers were fine-tuned during training.

Each 224x224 joint-centered crop was first tokenized by a 14x14 convolutional patch-embedding layer with 384 output channels. The resulting patch tokens were processed through 12 transformer encoder blocks consisting of hidden dimension 384, 6 attention heads (head dimension 64), MLP hidden dimension 1536, GELU activation, pre-LayerNorm with LayerScale. The final 384-dimensional CLS token generated by the backbone was projected to a 2-dimensional image feature vector using a fully connected layer.

In parallel, a 13-dimensional tabular vector was constructed using binary tenderness status, binary swelling status, and an 11-way one-hot encoding of joint type (1st CMC joint, 1st MCP joint, 1st IP joint, and 2nd–5th MCP and PIP joints). This tabular vector was processed through a fully connected layer to generate 2-dimensional clinical feature vector.

The image feature vector and clinical feature vector were concatenated into a 4-dimensional fused representation and passed through a final fully connected layer (input dimension 4, output dimension 1), followed by a sigmoid activation function to generate the per-joint probability of US-detected inflammation.

2.4 Training strategy

Binary cross-entropy loss was used as the objective function for model optimization. The model was trained for 300 epochs with a batch size of 64, using the AdamW optimizer (learning rate = 5×10^{-4}, weight decay = 1×10^{-5}). Validation loss was monitored at every epoch, and the model state corresponding to the lowest validation loss was retained as the final checkpoint, effectively implementing early stopping.

2.5 Dataset split and validation strategy

A temporal split strategy was used for model development and evaluation. Final model was trained on Dataset 2 with internal validation and subsequently evaluated on the temporally held-out Dataset 3.

2.6 Implementation environment

The model was implemented in PyTorch using the Hugging Face transformers DINOv2 implementation and conducted on the Pegasus high-performance computing cluster equipped with NVIDIA V100 GPUs (16 GB memory). The software environment was based on Python 3.10 and included commonly used scientific libraries such as PyTorch, torchvision, NumPy, pandas, OpenCV, seaborn, and scikit-learn.
